# Supplementary material for: Chrono-Nutritional Patterns, Medical Comorbidities, and Psychological Status in Patients with Severe Obesity
Source: Nutrients. 2023 Dec 3;15(23):5003. doi: 10.3390/nu15235003 (PMC10707777; doi:10.3390/nu15235003)
Supplement: Supplementary file 1 [file nutrients-15-05003-s001.zip › nutrients-2698213-supplementary.pdf]

Supplementary materials

Figure S1. Example of chrono-nutritional time series for a patient (top) and estimated probability of a meal during the day (bottom)

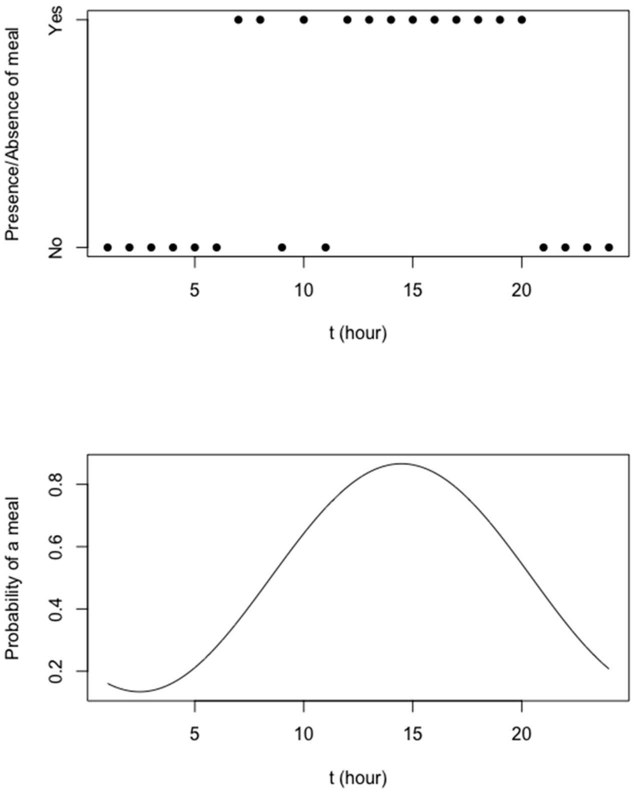

Table S1. Main characteristics overall (N= 173) and according to the 4 estimated chrono-nutritional profile.

**Characteristics**	**Chrono-nutritional profiles**				**p-value**[2]
	**1**, N = 80[1]	**2**, N = 11[1]	**3**, N = 55[1]	**4**, N = 27[1]	
**Gender, Female**	24 (30%)	2 (18%)	24 (44%)	6 (22%)	0.15
**Age (years)**	45 (39, 53)	49 (44, 53)	48 (41, 56)	48 (33, 53)	0.27
**Educational level**					0.71
Middle school	34 (43%)	5 (45%)	21 (38%)	12 (44%)	
High school	34 (43%)	6 (55%)	26 (47%)	9 (33%)	
Degree or higher	12 (15%)	0 (0%)	8 (15%)	6 (22%)	
**Physical activity**	16 (20%)	2 (18%)	7 (13%)	4 (15%)	0.72
**Alcohol consumption**	10 (13%)	2 (18%)	10 (18%)	3 (11%)	0.74
**Smoking habit**	18 (23%)	5 (45%)	8 (15%)	2 (7.4%)	0.038
**BMI category**					0.45
[32.9,40.2]	32 (40%)	1 (9.1%)	16 (29%)	9 (33%)	
(40.2,46.2]	22 (28%)	5 (45%)	20 (36%)	10 (37%)	
(46.2,80.3]	26 (33%)	5 (45%)	19 (35%)	8 (30%)	
**Shift work**	10 (13%)	3 (27%)	13 (24%)	6 (22%)	0.24

[1] Median (IQR) or Frequency (%)

[2] Fisher's exact test; Kruskal-Wallis rank sum test

Table S2. Clinical outcome and psychological score according to the 4 estimated chrono-nutritional profiles.

**Characteristics**	**Chrononutritional profiles**				**p-value**[2]
	**1**, N = 80[1]	**2**, N = 11[1]	**3**, N = 55[1]	**4**, N = 27[1]	
**Hypertension**	33 (41%)	5 (45%)	28 (51%)	10 (37%)	0.61
**Diabetes**					0.69
No	36 (45%)	2 (18%)	24 (44%)	10 (37%)	
Pre-diabetes	30 (38%)	6 (55%)	21 (38%)	13 (48%)	
Yes	14 (18%)	3 (27%)	10 (18%)	4 (15%)	
**Dyslipidemia**	46 (58%)	4 (36%)	38 (69%)	13 (48%)	0.11
**Use of Statins**	7 (8.8%)	0 (0%)	9 (16%)	2 (7.4%)	0.37
**CT (mmol/L)**	4.65 (4.06, 5.12)	4.86 (4.29, 5.59)	4.60 (4.03, 5.34)	4.43 (3.93, 5.00)	0.63
**HDL (mmol/L)**	1.17 (0.97, 1.37)	1.12 (1.08, 1.46)	1.16 (1.06, 1.40)	1.23 (1.00, 1.57)	0.89
**LDL (mmol/L)**	3.13 (2.62, 3.54)	3.22 (2.80, 3.57)	3.09 (2.42, 3.68)	2.85 (2.41, 3.35)	0.57
**TG (mmol/L)**	1.26 (0.91, 1.68)	1.28 (1.05, 2.34)	1.48 (1.12, 2.12)	1.07 (0.77, 1.54)	0.035
**HOMA**	4.0 (2.3, 6.2)	4.1 (2.9, 9.7)	4.7 (1.9, 6.9)	3.0 (2.0, 4.6)	0.45
**SCL90-R_GSI**	58 (50, 67)	50 (45, 59)	70 (53, 83)	64 (51, 83)	0.001
**SF-36 PH**	56 (39, 73)	58 (53, 69)	50 (27, 62)	46 (38, 68)	0.11
**SF-36 MH**	60 (46, 73)	59 (53, 77)	48 (36, 66)	56 (32, 68)	0.079
**Y-FAS score**	2.00 (1.00, 3.00)	1.00 (1.00, 3.50)	3.00 (2.00, 4.00)	3.00 (1.50, 5.00)	0.010
**EAT-26 score**	7 (3, 11)	10 (1, 12)	9 (4, 15)	10 (7, 17)	0.094
**BIS-11 score**	58 (52, 65)	56 (52, 62)	64 (55, 71)	62 (56, 67)	0.034
**BES score**	10 (4, 16)	8 (4, 15)	14 (9, 22)	18 (9, 24)	0.002

[1] Median (IQR) or Frequency (%)

[2] Fisher's exact test; Kruskal-Wallis rank sum test
